# The effect of trained parameters in Bayesian neural encoding models for the auditory system

**DOI:** 10.1186/1471-2202-15-S1-P67

**Published:** 2014-07-21

**Authors:** Eric Plourde

**Affiliations:** 1 Department of Electrical and Computer Engineering, Université de Sherbrooke, Sherbrooke, Quebec, J1K 2R1, Canada

## 

Bayesian neural decoding models aim at estimating an extrinsic stimulus, such as speech, from a neural population. They have been used to study several physiological systems such as the motor or auditory systems [1]. Such decoding models require the use of an encoding model, i.e. which aim at estimating the neural spikes from the stimulus [2]. One encoding model that has been used extensively in the past models the instantaneous spiking rate of a neuron using a generalized linear model (GLM). When applied to the auditory system, this GLM has three parameters that account respectively for 1) the spontaneous firing rate of the decoded neuron, 2) the spectro-temporal receptive field of the neuron and 3) the intrinsic dynamics of the neuron, such as refractory periods, bursting and network dynamics [3]. In a decoding framework, these parameters are estimated by fitting the encoding model to spike trains measured when presenting a specific stimulus (the training set) and used afterwards to perform the decoding of spike trains obtained when presenting a different stimulus (the test set). While it could be assumed that the parameters associated with the spontaneous rate and receptive fields won’t change significantly for different stimuli for a given neuron, the intrinsic dynamics will most likely change. Here we wish to investigate how trained GLM parameters, i.e. fitted for some specific stimulus, of a neural encoding model for the auditory system can accurately represent the spikes measured for a different stimulus. To do so we use a goodness of fit metric, the normalized Kolmogorov-Smirnov (KS) statistic; a normalized KS < 1 indicating an excellent fit. Table 1 presents the normalized KS statistic obtained when evaluating the goodness of fit of the encoding model using trained parameters on measured spikes from the test set with 2-fold cross-validation. The recordings are from 54 auditory nerve neurons (20 trials each) in anesthetized cats. We observe that the normalized KS values are < 1 for only 13% (7/54) of the neurons suggesting an extremely poor fit of the encoding model. Using such encoding model parameters in a decoding framework will therefore introduce a strong bias in the decoded stimulus that is not currently taken into account in neural decoding models.

**Table 1:** Number of neurons for which the maximum normalized KS statistics using 2-fold cross validation is within a given range for both training and test sets. A KS statistic < 1 indicates an accurate fit of the model. (*n*=54, 20 trials each, recordings from the auditory nerve of anesthetized cats)

Range of maximum normalized KS statistic, *nKS*	Number of neurons with *nKS* within the given range	
	
	Training set	Test set
0 <*nKS* ≤ 1	54	7

1 <*nKS* ≤ 2	0	9

2 <*nKS* ≤ 3	0	13

3 <*nKS* ≤ 4	0	12

*nKS* > 4	0	13
